# Accurate Monitoring of 24-h Real-World Movement Behavior in People with Cerebral Palsy Is Possible Using Multiple Wearable Sensors and Deep Learning

**DOI:** 10.3390/s23229045

**Published:** 2023-11-08

**Authors:** Ivana Bardino Novosel, Anina Ritterband-Rosenbaum, Georgios Zampoukis, Jens Bo Nielsen, Jakob Lorentzen

**Affiliations:** 1 Department of Pediatric Neurology 5003, University Hospital Copenhagen, Rigshospitalet, 2100 Copenhagen, Denmark; j.lorentzen@sund.ku.dk; 2 Department of Neuroscience, Faculty of Health and Medical Sciences, The Panum Institute, Copenhagen University, 2200 Copenhagen, Denmark; georgios.zampoukis@sund.ku.dk (G.Z.); jbnielsen@sund.ku.dk (J.B.N.); 3 The Elsass Foundation, 2920 Charlottenlund, Denmark; arr@elsassfonden.dk

**Keywords:** cerebral palsy, movement behavior, wearable sensors, deep learning, monitoring

## Abstract

Monitoring and quantifying movement behavior is crucial for improving the health of individuals with cerebral palsy (CP). We have modeled and trained an image-based Convolutional Neural Network (CNN) to recognize specific movement classifiers relevant to individuals with CP. This study evaluates CNN’s performance and determines the feasibility of 24-h recordings. Seven sensors provided accelerometer and gyroscope data from 14 typically developed adults during videotaped physical activity. The performance of the CNN was assessed against test data and human video annotation. For feasibility testing, one typically developed adult and one adult with CP wore sensors for 24 h. The CNN demonstrated exceptional performance against test data, with a mean accuracy of 99.7%. Its general true positives (TP) and true negatives (TN) were 1.00. Against human annotators, performance was high, with mean accuracy at 83.4%, TP 0.84, and TN 0.83. Twenty-four-hour recordings were successful without data loss or adverse events. Participants wore sensors for the full wear time, and the data output were credible. We conclude that monitoring real-world movement behavior in individuals with CP is possible with multiple wearable sensors and CNN. This is of great value for identifying functional decline and informing new interventions, leading to improved outcomes.

## 1. Introduction

Cerebral palsy (CP) is a clinical diagnosis describing a disturbance of the developing fetal or early infant brain caused by non-progressive injuries or abnormalities [1]. Registries from European countries show a prevalence of 2–3‰ for live births [1]. The latest study in Denmark suggests a prevalence of 1‰ for live births in children born at term [2]. In most cases, CP causes disorders in motor development characterized by various abnormal patterns of movement and posture related to impaired coordination of movements and regulation of muscle tone [3]. The implications are limitations in activity level and societal participation that persist throughout the lifespan. The Gross Motor Function Classification Scale (GMFCS) [4] categorizes the physical abilities of individuals with CP into five levels, ranging from independent ambulation in level I to complete dependency and limited movement control in level V. Although CP is non-progressive, many individuals experience motor ability deterioration during adolescence. Research suggests a decrease in gross motor abilities might commence before age 7 [1,5].

In the clinical or hospital setting, healthcare professionals utilize a range of observations and measurements to assess and manage the health and physical capabilities of individuals with CP. However, whether these assessments can accurately reflect real-world behaviors involving cognitive exertion and where emotions and environmental factors influence physical abilities remains unclear. There has been an increasing interest in wearable technologies in healthcare and behavioral research to overcome this challenge. Most available wearables track real-world physical activity (PA) and sedentary behavior, and research shows that children, adolescents, and adults with CP have significantly reduced PA levels compared to their typically developed peers [6,7]. Further, children with CP spend significantly more time sedentary during waking hours [8], and very few adhere to 24-h activity guidelines for children with CP [9]. This can be part of the reason behind the higher incidence of lifestyle-related diseases among adults and elderly individuals with CP [10,11]. While PA and sedentary behavior measurements are helpful, they offer a partial understanding of movement behavior. By observing the movement behaviors of individuals with CP in their daily lives, significant insights can be gained into their functional decline. Identifying the specific extremities involved and their contributions is crucial beyond simply measuring PA and sedentary behavior. These insights can inform the development of effective interventions and management strategies for individuals with CP. To our knowledge, only a few studies use multiple wearables and can thus describe a spectrum of movement behaviors in a CP population [12,13,14,15,16,17]. Even fewer studies can describe the movement behavior of individuals with severe CP disabilities [18,19]. Although wearable technology shows excellent potential for monitoring real-world behavior, its success relies on dependable technology and user willingness. To our knowledge, very few large studies [9] reporting real-life activity behaviors in the CP population have 24 h of data. Valid days are as low as 8 [20] and 10 h of recording per day [21].

To promote health and guide novel intervention development, we need better insight into how real-world movement behaviors are impacted and changed in people with CP across GMFCS levels. Deep learning is a machine learning technique that is widely used in human activity recognition. This approach has been shown to be efficient in learning representative features of movement from raw signals and has the ability to act as universal function approximators, given a large enough network and sufficient observations [22]. Several studies have explored the application of deep learning methods to monitor human movement behavior, particularly using kinematic data from wearable sensors. Recent reviews have identified advances, challenges, and opportunities for future deep learning algorithms in this field [22,23]. However, the challenge often transcends the algorithms themselves but pertains to the entire process of collecting and analyzing data. This involves overcoming technical limitations and ensuring a reliable and user-friendly approach. While existing studies have made strides in this area [24], the need for a process that is both simple and effective while ensuring accurate results over extended periods in real life remains significant. For individuals with CP, this need is further accentuated, as their movement patterns and capabilities vary greatly, requiring the use of multiple sensors. Collecting and analyzing data from sensors can be challenging. Motion sensors, while invaluable, are not without their drawbacks, such as short battery life, limited wireless range, and difficulties in synchronizing data from multiple sensors. These challenges can hinder the creation of a reliable process for recognizing movement behavior over extended periods and from multiple synchronous sensors.

The novelty of this study lies in the development of an end-to-end process for reliably collecting data over extended periods and optimizing a custom deep learning architecture. We have combined the best features of established classification architectures such as Residual Networks (ResNets) and Visual Geometry Groups (VGGs). This custom architecture has been designed with a strong focus on performance, execution speed, and accuracy. Moreover, the network has been built to be extendable. The technical novelty lies in the encoding of the data in 3-dimensional arrays (2-channel image) and the use of a 2-dimensional Convolutional Neural Network (CNN), which offers a greater receptive field. The network has been trained to recognize extremity movements, posture, and walking. We aim toward home therapy compliance monitoring, real-time movement behavior feedback, control of therapy interventions, and monitoring behavioral changes throughout all CP GMFCS levels. To build knowledge for future studies of movement behavior in CP, the current study assesses the developed network’s performance and evaluates the feasibility of 24-h recordings in a real-world setting.

## 2. Materials and Methods

Using accelerometer and gyroscope data, a custom image-based deep learning CNN was modeled and trained by GZ.

A primarily cross-sectional cohort of 14 typically developed adults and one adult with CP was recruited from online and offline advertising at the University of Copenhagen, Denmark.

The CNN predictions were assessed against both timestamped test data as well as against video annotations from two independent human annotators (ARR and IBN). The video data comprised 70 min recordings of 14 typically developed adults at the Department of Neuroscience, University of Copenhagen, Denmark.

The feasibility of 24-h recordings in a real-life context was tested on one typically developed adult from the cohort and one adult with CP.

### 2.1. Description of Labels

CP affects different body parts (e.g., unilateral, bilateral, diplegia, and quadriplegia) [1]; hence, we found it relevant to classify right and left upper- and lower extremity movement. Further, people with CP present different mobility abilities, making the classification of postures as lying, sitting, and standing, as well as the activity of walking, relevant.

**Figure 1:**
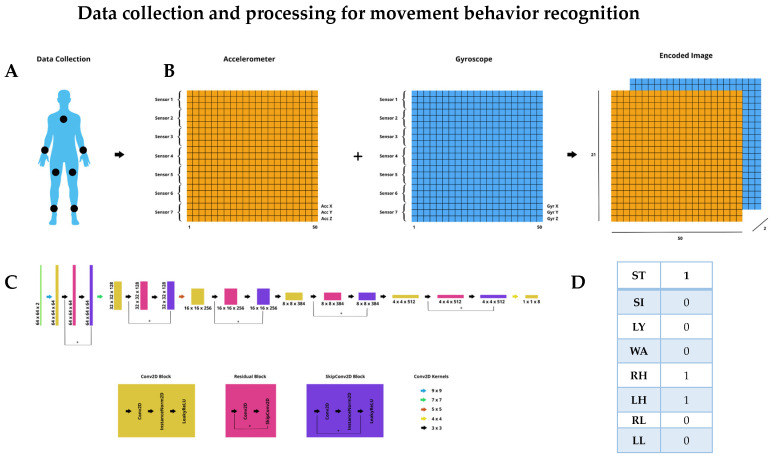
Data collection, processing, and encoding. (**A**) Sensor placement; (**B**) 2-channel image creation from the accelerometer and gyroscope data; (**C**) Network schematics; (**D**) Label classification; ST, standing; SI, sitting; LY, lying; WA, walking; RH, right hand; LH, left hand; RL, right leg; LL, left leg.

To allow for the classification of the labels mentioned above, participants wore seven sensors, symmetrically attached to wrists, lower legs, thighs, and one on the sternum (Figure 1A). 

### 2.2. Description of Sensors

We sought battery-operated wireless sensors measuring and reporting acceleration (accelerometer) and angular rates (gyroscope), which were non-intrusive, lightweight, and wearable. Further, we needed sensors to be easily mounted on a person.

Movesense HR2 (Suunto, Vantaa, Finland) is a waterproof sensor used for CNN performance testing. Data were streamed via Bluetooth to an iOS mobile data logger application (Movesense showcase App, Suunto, Vantaa, Finland). For the 24-h recordings, we predicted a risk of participants unintentionally moving outside the Bluetooth connection range. Hence, protocol dictated that the iOS mobile be worn in an arm sleeve. However, during the pilot testing of 24-h recordings, we experienced data loss as body parts could block Bluetooth signals from sensors, resulting in sensor drop. Problems with Movesense sensor drop have previously been described [25]. Therefore, we switched to a different sensor for the 24-h recording. MetaMotions (Mbientlab, San Francisco, CA, USA) has 512 MB NAND Flash onboard memory and was consequently used for 24-h recordings, omitting the iOS phone and arm sleeve from the protocol. Data were transferred to the Metabase application. MetaMotions is not waterproof.

Both brands of sensors provided CNN with the same kinematic data and were worn in the same bodily locations. Movesense HR2 is in a round case, measuring 3.7 cm in diameter, 1.1 cm thick, and weighing 10 g with a battery. MetaMotions is in a semi-rectangular case, is 2.7 cm wide, 0.4 cm thick, and weighs 7.6 g with a battery.

### 2.3. Data Collection

Data are acquired from seven sensors, where individual sensors collect data. The accelerometer detects and measures three-axis linear acceleration, e.g., x, y, and z, while the gyroscope detects and measures three-axis angular velocity, e.g., roll, pitch, and yaw. We will also refer to the latter as x, y, and z for convenience. The result is a unified sample from each sensor, which consists of six components: Acc X, Acc Y, Acc Z, Gyr X, Gyr Y, and Gyr Z. The maximum measuring range for the accelerometer was set at ±8 g and for the gyroscope at ±1000 deg./s. A sampling rate of 50 Hz is used.

### 2.4. Data Processing and Encoding

A brief overview of the CNN architecture is provided below. A more detailed description can be found in Appendix A.

To train our model, we used a supervised learning approach. Our dataset was created by timestamped scripted movements from one typically developed adult (GZ). We used a Python script to signal a range of extremity movements during lying, sitting, standing, or walking, covering different label combinations. This created a balanced and diverse dataset consisting of 10,000 one-second samples. Since the movements were timestamped alongside the sensor recordings, we eliminated the need for manual annotation and were able to create a dense dataset quickly. The network was trained using 90% of the data representing the ground truth, while the remaining 10% was used as test data.

As CNN accepts images as input, sensor data are preprocessed for the imaging of signals. Fifty samples (1-s window) from each of the seven sensors are taken, separating the accelerometer and gyroscope data. A 2-channel image is then created, with the first channel holding all the accelerometer data and the second channel holding all the gyroscope data. Each pixel row contains the corresponding sub-sensor components (x, y, z) for each of the seven sensors, and each pixel column is an individual sample. The result is an image of resolution 50, 21, 2 (50 samples, seven sensors times 3 components, 2 channels). The process is illustrated in Figure 1B.

The CNN consists of custom-made convolutional layers (Figure 1C), on which features are extracted and data are classified into prespecified labels (Figure 1D). Extremity movement, posture, and walking are described as binary variables having only one of two states: 0 and 1, where 0 means that the label is absent and 1 means that it is present.

### 2.5. Sensor and Video Recordings

Typically developed adults were recorded on video while wearing seven Movesense HR2 sensors in a laboratory setting. Sensors were worn as previously described and attached with MEDMAX CGM skin adhesive patches. Sensory and video recordings were performed simultaneously over five minutes to allow for later human annotations. The test facility was a large gait lab equipped with treadmills, a stationary bike, storage facilities, stairs, a whiteboard, consoles and desk space, a therapist bench, and chairs, but also with enough space to walk around in.

First, participants were guided by an instruction video requiring them to mirror two minutes of activities with the upper and lower extremities while sitting, standing, and walking in place. Immediately thereafter, they completed 3 min of free activity using the whole test facility. Participants were informed that they could use the space, equipment, and furniture however they wanted, and the more variety they showed in their activity behavior, the better it would be for data collection.

Two researchers were present during the recordings. One (GZ) continuously checked that data were transferred correctly from individual Movesense sensors to the data logger via Bluetooth. One researcher (IBN) followed participants and video-recorded them on an iOS phone.

### 2.6. Video Annotation Protocol

Video data were annotated through the 6.0 version of Anvil Annotation Software (185 Green End Road, Cambridge, UK).

**Scheme 1:**
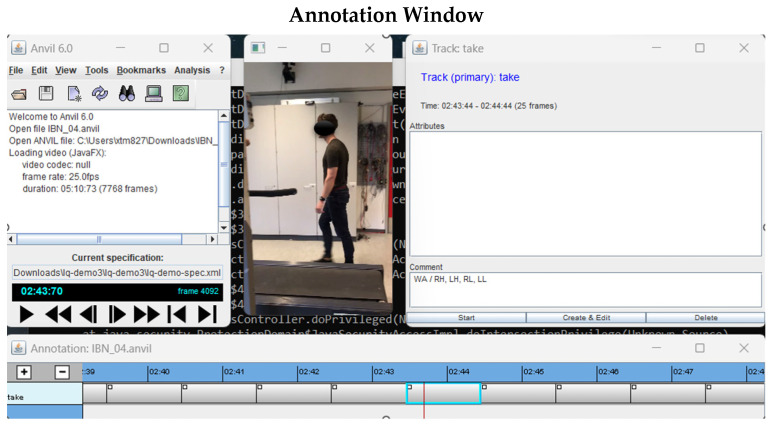
View of the Anvil annotation window displaying annotations with synchronized video data.

To ensure a reliable assessment of labels, two researchers (IBN and ARR) annotated each video independently. Annotations were made every second in a 3-s window to accurately distinguish between standing and walking. Further, the protocol stated no labeling if a limb was in movement for less than three frames. If the activity was not represented in the predefined labels, it would be marked with a question mark and left out of the later analysis. The coding scheme consisted of walking (WA), standing (ST), sitting (SI), and lying (LY). Extremity movement was separated from walking and posture by a forward slash (/) and coded right hand (RH), left hand (LH), right leg (RL), and left leg (LL) (Scheme 1).

Before video annotation, annotator training was undertaken, including studying the protocol, learning predefined labels, and conducting one annotation of a pilot video together, including a discussion of differences until consensus was reached.

### 2.7. 24-h Recordings in a Real-World Context

Before the 24-h recordings, we charged each MetaMotions sensor to its total capacity. The sensors were symmetrically attached, as previously described (Figure 1A), with skin adhesive patches. For the typically developed adult, it occurred in the gait lab, and for the adult with CP, it took place at home. Recordings were terminated at least 24 h after initiation. Participants were free to participate in any activity except swimming or bathing.

Immediately after recordings, participants were asked about adverse events, defined as shear, pressure soars, and skin irritation. Twenty-four recordings were considered feasible if data collection was continuous and sensors were worn and recorded for the full wear time.

We reviewed data from 24-h recordings to examine the credibility of network output using the following criteria: The posture labels and walking labels are mutually exclusive. Hence, only one posture, or walking, can be classified at a given time. All four extremity movements can co-occur. During lying, sitting, and standing time, extremity movements may either be present or absent. In walking time, at least both lower extremity labels must be present during a one-second window.

### 2.8. Data Analysis

Because of the possibility of agreement occurring by chance, we use Cohen’s kappa coefficient (κ) to calculate inter-annotator agreement. (κ) is interpreted as: 0.0–0.20, none; 0.21–0.39, minimal; 0.40–0.59, weak; 0.60–0.79; 0.80–0.90, strong; >0.90, almost perfect agreement [26].

We evaluated the performance of CNN on two fronts: against timestamped and scripted test data and human video annotations.

Accuracy was used to determine how often the CNN made a correct prediction across the entire dataset. To determine the similarity between the network’s positive prediction outputs, test data, and human annotations of the videos, we calculated the Intersection over Union (IoU). IoU is framed in the following formula: TP / (TP+FP+FN) where TP is the true positive, FP is the false positive, and FN is the false negative.

We created multilabel confusion matrix comparisons showcasing TP, FP, true negative (TN), and FN rates.

To compare the networks’ ability to discriminate between the absence and presence of the different labels, we used the Area Under the Receiver Operating Characteristic Curve (AUROC). An AUROC of 0.5 corresponds to a coin flip; <0.7 is suboptimal performance; 0.70–0.80 is good performance; >0.8 is excellent performance.

The CNN comparisons with test and annotation data and all classification evaluation metrics were performed in Python 3.11.0 using the TorchMetrics library.

## 3. Results

### 3.1. Performance against Test Data

The CNN demonstrated exceptional performance when tested against the timestamped scripted test data, with a mean accuracy of 99.7%. Its general sensitivity (TP) and specificity (TN) were 1.00, and the mean IoU was 0.99. The multilabel confusion matrix can be found in Appendix B.

### 3.2. Performance against Human Video Annotations

Fourteen healthy adults completed five minutes of scripted and free movement with annotations for each second, amounting to 4200 data points. Activities not represented in predefined labels were crawling, jumping, rolling, bending forward, cartwheeling, kneeling, running, stair climbing, cycling, and planking. These activities represented 6% of the data points.

Inter-annotator agreement was (κ) 0.89, indicating trustworthy annotations and that labels are firmly described.

CNN’s mean accuracy was 83.4%. The general sensitivity (TP) is 0.84, and the specificity (TN) is 0.83. The mean IoU is 0.68, meaning 2/3 of labels are predicted correctly.

**Figure 2:**
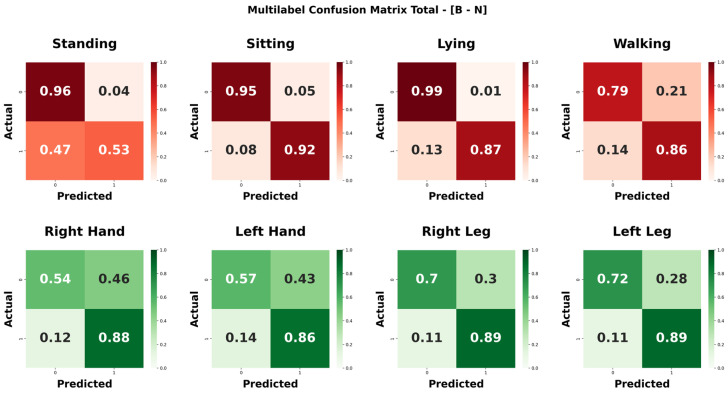
Multilabel confusion matrix between human annotations (actual) and network output (predicted).

The multilabel confusion matrix shows that the network performs best on posture sitting, lying, and walking compared to human annotations (Figure 2). Noteworthy is the network’s underprediction of standing (FN 0.42) and overprediction of walking (FP 0.21). Most disagreements were found for upper- and lower-limb activity, where the network predicted more extremity movement than the annotators (FP 0.28–0.49).

**Figure 3:**
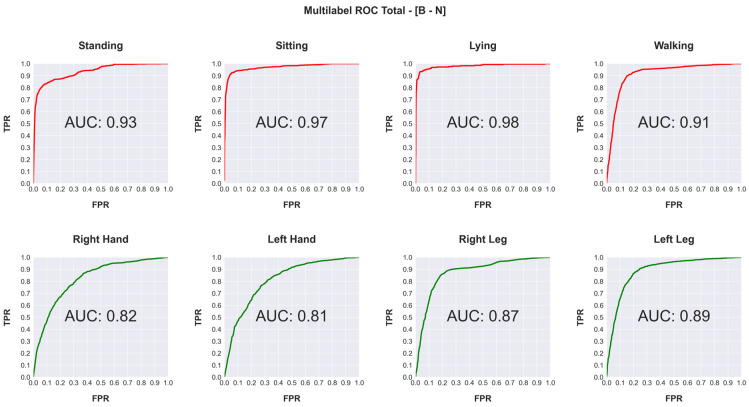
Multilabel area under the receiver operant curve. TPR, true positive rate; FPR, false positive rate.

AUROC at 0.81–0.98 (Figure 3) shows excellent discrimination between TP and TN rate ability against human video annotations at the current operant condition (Figure 3).

### 3.3. Feasibility of 24-h Recordings

Subjects were one typically developed man and one woman with left-sided CP, GMFCS I.

Recordings were continuous throughout >24 h for both subjects (Table 1), and sensors were worn for the full wear time. There were no reported adverse events.

We found the data credible, with no instances of more than one posture or walking occurring concurrently, and that at least both lower extremity labels were present in a one-second window during the walking time.

Mapping and visualization of posture transitions, the absolute and relative percentage of extremity usage, the extremity usage timeline, and the posture timeline can be found in Appendix C.

Of interest in the 24-h data are the noteworthy disuse of the left upper extremity relative to right upper extremity usage in the person with left-sided CP.

## 4. Discussion

We have presented a comprehensive end-to-end process for the accurate collection of movement behavior data over a 24-h period. This process involved encoding data in a format that is scalable and optimizing a custom deep learning architecture tailored to recognize postures lying, sitting, and standing, walking activity, and extremity movement in humans. The network performed exceptionally well when tested against the timestamped scripted movement, with a mean accuracy of 99.7%, TP and TN at 1.00, and a mean IoU of 0.99. Against human video annotation, the network performed at a high 83.4% mean accuracy, TP 0.84, and TN 0.83. Recordings >24 h in real-world contexts were successful without data loss or adverse events.

We have explored the performance of CNN against human video annotations with several metrics. The mean accuracy metric provides a label-to-label representation of the percentage agreement between the network and annotations; although easily understandable, it does not reveal the nature of disagreements. The IoU of 0.68 provides an accurate representation of the TP correctly detected by the network. We further explored accuracy with the multilabel confusion matrix and found that the network overpredicts extremity movement (FP 0.28–0.49), standing is underpredicted (FN 0.42), and walking is overpredicted (FP 0.21). When interpreting these results, it is paramount to consider whether inaccuracies are built into the network or if there are inaccuracies in human annotations. This study followed an annotation protocol to minimize inconsistencies, and two independent annotators underwent annotator training. Inter-annotator agreement was strong, (*κ*) 0.89, indicating that annotations were trustworthy and labels easily understandable. There is, however, a possibility of poor-quality video data, e.g., out-of-camera events. The gait lab where video recordings took place was fully equipped as described, and participants were free to move in and around equipment that could hide movements from the camera and, thus, annotators. Further, participants were recorded via handheld iOS phones. As the movement was free, participants were moving in unpredictable patterns, allowing for body positioning relative to the camera, hiding extremity movements. This would cause a correct network prediction to be marked as false, represented by CNN’s overestimation of extremity movements.

Compared to human annotators, the network underpredicted standing and overpredicted walking. Both the network and the annotators used a 1-s window to judge labels. However, this window length would have been inadequate for human annotators to accurately differentiate between standing while moving legs and walking. To mitigate this issue, annotators were given a 3-s window to better judge the second in question. Determining whether a participant is standing, moving legs, or walking is rather difficult for human annotators. CNNs have performed expertly in various medical fields [27] and human activity recognition [25]. We argue that inaccuracies when comparing this CNN prediction with annotations were likely due to the network outperforming human annotators.

Obtaining large amounts of high-quality, balanced, labeled data for model training and optimization is costly and time-consuming [23,27]. Considering this and the potential inaccuracies in human annotations, the training data for the CNN was timestamped and scripted. Although this might question the network’s applicability in real-world contexts and for individuals with CP, we maintain that the network is applicable. The labels are simple and commonly encountered in everyday life, and even though there is a great deal of variation in how a particular activity can be performed, our labels have distinct characteristics. The CNN employs predictive algorithms based on acceleration, orientation, and angular velocity analysis. This universal kinematics applies to all humans, regardless of whether they are typically developed or have neurological impairments. Furthermore, data are obtained by seven wearable sensors that provide both accelerometer and gyroscope data, which receive better results than one particular sensing modality alone [23].

Our research has shown that the CNN and seven wearable sensors can provide continuous and credible data for 24 h without adverse events. The 24-h feasibility testing holds significant importance in the development of our method, shedding light on the critical need for onboard sensor memory to combat Bluetooth signal interference during real-world movement behavior monitoring. Moreover, a data-steaming process requires participants to use a smartphone attached to their body, which can cause discomfort during longer recording periods. We achieved an optimized data collection process by integrating MetaMotions sensors capable of both local data recording and wireless streaming without Bluetooth Low Energy bandwidth limitations. This integration also successfully removed the bottleneck associated with the number of sensors used simultaneously. Another notable finding during the 24-h recordings was that the individual with left-sided CP exhibited a higher frequency of right extremity usage than the typically developed individual. This supports the potential for using the method for monitoring movement behavior in the population with CP. Although we acknowledge the limitations of our small feasibility sample size, this research is an essential preliminary step toward evaluating the method’s potential in a larger sample of individuals with CP. The limitations of our present CNN model for real-world movement behavior recognition must also be acknowledged. Currently, CNN is not trained to differentiate between self-generated movement and movement caused by external factors. As such, it is essential for future research to consider GMFCS levels and supplement objective measurements of movement behavior with contextual information.

## 5. Conclusions

In conclusion, our study presents a promising method for accurately detecting movement behavior in individuals with CP in real-world situations. The use of seven wearable sensors and the custom-made CNN resulted in exceptional performance, surpassing human annotation and continuous and providing credible data for 24 h without adverse events. This approach has the potential to enhance our understanding of real-world movement behaviors in individuals with CP beyond measuring physical activity and sedentary behavior. Further, there is potential to improve the detection of functional decline. Our findings provide a strong foundation for future research that could lead to real-time movement behavior feedback and improved outcomes for individuals with CP.

## Figures and Tables

**Table 1:** TR, total recording time; ST, standing; SI, sitting; WA, walking; LY, lying; RH, right hand; LH, left hand; RL, right leg; LL, left leg.

Data from 24-h Recordings										
		Absolute Posture and Walking					Relative Limb Usage			
Subject	TR Time	ST (%)	SI (%)	WA (%)	LY (%)	Transitions	RH (%)	LH (%)	RL (%)	LL (%)
TD	24 h 48 m 23 s	12.7	50.3	1.7	35.4	944	33.1	24.4	21.2	21.3
CP	24 h 01 m 49 s	15.8	38.2	3.0	42.9	1212	52.2	22.7	11.7	13.4

**Figure A1:**
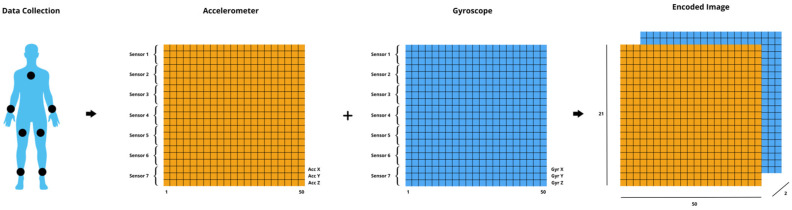
Encoded Image.

**Figure A2:**
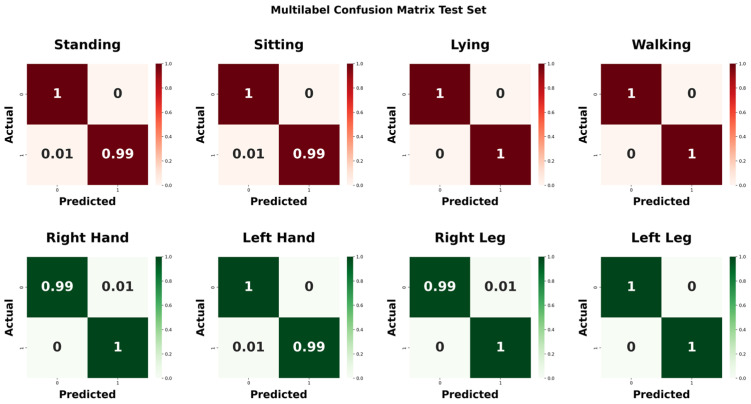
Multilabel confusion matrix between test data (actual) and network output (predicted).

**Figure A3:**
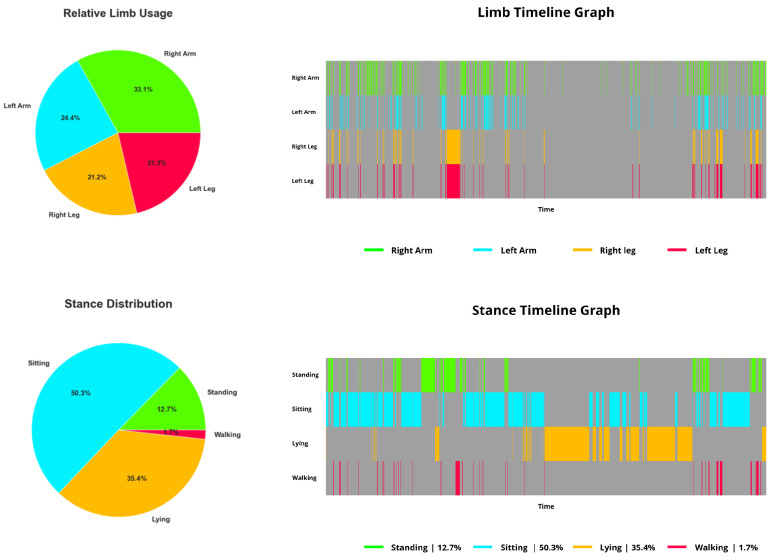
Visual representation of the 24-h movement behavior of a typically developed man.

**Figure A4:**
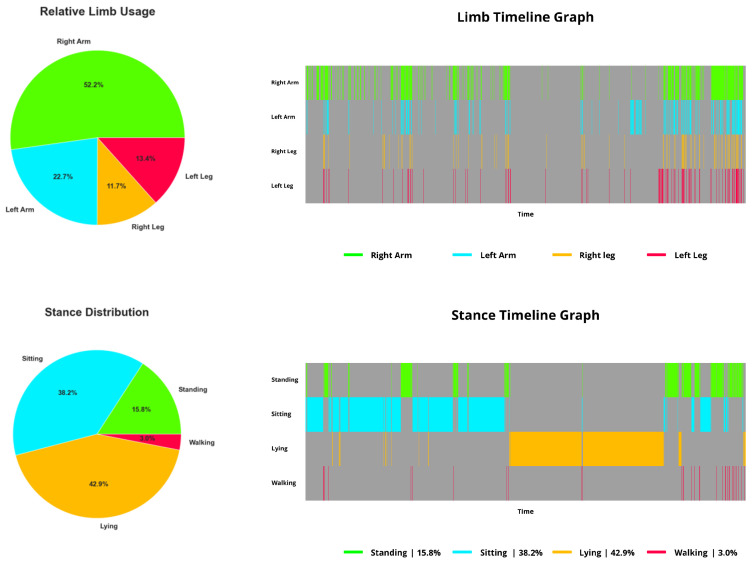
Visual representation of the 24-h movement behavior of a female with cerebral palsy.

## Data Availability

The data presented in this study are openly available at github.com/georgezampoukis/movement-and-posture-classification.

## Appendix A

The network is a deep Convolutional Neural Network (CNN) for image classification tasks. It is characterized by using residual blocks, skip connections, and instance normalization to enhance the learning process. It is a custom architecture that combines elements and ideas from well-known and established networks such as ResNet, VGG, and InceptionV3.

### Appendix A.1. Key Components

#### Appendix A.1.1. Strided Conv2D

Strided Conv2D is a custom 2D convolutional layer with a stride of 2, followed by instance normalization and the LeakyReLU activation function. We perform this operation with a stride of 2 (except the first one) to gradually reduce spatial dimensions while increasing the feature pool. Kernel sizes are gradually descending, from 9 to 3, to enable the network to focus more on high-frequency details as the feature pool increases. After the convolution, instance normalization and LeakyReLU activation with a negative slope of 0.2 are applied to the output feature maps to improve convergence and counteract vanishing gradients.

#### Appendix A.1.2. SkipConv2D

SkipConv2D is a special 2D convolutional layer that incorporates skip connections. These connections combine the feature maps from before and after using residual blocks, which can help maintain gradient flow and promote faster convergence.

#### Appendix A.1.3. Residual Block

A residual block is a building block consisting of two Conv2D layers. The input is added to the output of the second Conv2D layer, creating a skip connection that helps maintain gradient flow through the network. This design choice enables the network to learn more complex features and improves its generalization ability.

#### Appendix A.1.4. Dropout2D

Dropout2D is employed to reduce overfitting and improve the generalization capability of the architecture. The dropout rate is configurable, with a default value of 0.5.

#### Appendix A.1.5. InstanceNorm2D

For normalization layers, InstanceNorm2D was chosen as it best fits the encoded 2-channel image, allowing for faster convergence and optimal weight normalization.

#### Appendix A.1.6. FCN Approach

The network follows a fully convolutional approach (FCN), consisting only of convolutional layers and no linear/dense layers. While most classification networks rely on dense layers for the classification part, we found, after experimentation, that a fully convolutional approach yielded slightly better results. This approach is highly beneficial as it preserves spatial information throughout the whole network while significantly improving model efficiency by drastically decreasing the number of trainable parameters.

### Appendix A.2. Implementation and Training

The model and the training logic were implemented in Python 3.11.0 using the Pytorch framework. An initial learning rate of 1 × 10^−4^ was used and gradually decayed to 5 × 10^−6^ to achieve the lowest possible loss (Binary Cross Entropy). We make use of the Adam optimizer with β1 = 0.9 and β2 = 0.999. The batch size was set to 16, and the train-validation split was set to 80 and 20, respectively. The model achieved the best loss after 50 training epochs, or 25,000 total steps for a dataset of 10,000 samples.

### Appendix A.3. Data Processing and Encoding

The data from each Mbient sensor are stored in a CSV format, representing a time series of x, y, and z data from each sub-sensor (accelerometer and gyroscope). We use a 1-s timeframe as our classification window and record data from the sensors with a sampling rate of 50 Hz. As the 2D convolution network was designed to accept 2-channel images (3D arrays) as input, the data from the sensors must be encoded in such an image that would effectively represent 1 s worth of data from seven sensors. To achieve this, we first slice 50 samples (1 s of data) from each component (x, y, z) from each sub-sensor (accelerometer and gyroscope) from each of the seven sensors used for the recording. These samples are then concatenated vertically, as demonstrated in Figure 1, with all the accelerometer data in channel 1 and all the gyroscope data in channel 2, producing a final image of dimension: [2,7,21]—[Channels, Height, Width]. The channels effectively represent the sub-sensors (accelerometer and gyroscope > 2), the height represents the seven sensors (7 sensors ×3 components x, y, z > 21), and finally, the width represents the samples (1 s of samples at 50 Hz sampling rate > 50 samples). Lastly, the encoded 3D array is resized to 2 × 64 × 64 using nearest neighbor interpolation, producing the final encoded 2-channel image.

### Appendix A.4. Dataset

To train the network, a custom dataset was created by timestamped scripted movements consisting of 10,000 1-s samples. During this process, a Python script would indicate a range of movements to a performer, covering different label combinations every time to create a balanced and diverse dataset. Furthermore, since the indicated movements were timestamped in conjunction with the sensor recordings, we eliminated the need to manually annotate data, allowing a very dense dataset to be created quickly. This automated data production approach also allowed for quick prototyping of ideas since time was well spent manually annotating data. Furthermore, since the data that the sensors record is contained within a specific range (±10) and is already represented as float32 values, no data normalization was applied.
